# Performance of ChatGPT, human radiologists, and context-aware ChatGPT in identifying AO codes from radiology reports

**DOI:** 10.1038/s41598-023-41512-8

**Published:** 2023-08-30

**Authors:** Maximilian F. Russe, Anna Fink, Helen Ngo, Hien Tran, Fabian Bamberg, Marco Reisert, Alexander Rau

**Affiliations:** 1 Department of Diagnostic and Interventional Radiology, Medical Center - University of Freiburg, Faculty of Medicine, University of Freiburg, Breisacher Str. 64, 79106 Freiburg, Germany; 2 Department of Stereotactic and Functional Neurosurgery, Medical Center - University of Freiburg, Faculty of Medicine, University of Freiburg, Freiburg, Germany; 3 Medical Physics, Department of Diagnostic and Interventional Radiology, Medical Center - University of Freiburg, Faculty of Medicine, University of Freiburg, Freiburg, Germany; 4 Department of Neuroradiology, Medical Center - University of Freiburg, Faculty of Medicine, University of Freiburg, Freiburg, Germany

**Keywords:** Health care, Diagnosis, Medical imaging

## Abstract

While radiologists can describe a fracture’s morphology and complexity with ease, the translation into classification systems such as the Arbeitsgemeinschaft Osteosynthesefragen (AO) Fracture and Dislocation Classification Compendium is more challenging. We tested the performance of generic chatbots and chatbots aware of specific knowledge of the AO classification provided by a vector-index and compared it to human readers. In the 100 radiological reports we created based on random AO codes, chatbots provided AO codes significantly faster than humans (mean 3.2 s per case vs. 50 s per case, p < .001) though not reaching human performance (max. chatbot performance of 86% correct full AO codes vs. 95% in human readers). In general, chatbots based on GPT 4 outperformed the ones based on GPT 3.5-Turbo. Further, we found that providing specific knowledge substantially enhances the chatbot’s performance and consistency as the context-aware chatbot based on GPT 4 provided 71% consistent correct full AO codes for the compared to the 2% consistent correct full AO codes for the generic ChatGPT 4. This provides evidence, that refining and providing specific context to ChatGPT will be the next essential step in harnessing its power.

## Introduction

Accurate fracture classification substantially improves clinical decision-making while inaccurate classification can lead to inappropriate treatment strategies. The Arbeitsgemeinschaft Osteosynthese (AO) classification is an established system for fracture evaluation and adherence streamlines workflows and allows for optimized patient care, improved diagnostic accuracy and treatment planning, and ultimately better patient outcomes (https://www.aofoundation.org/).

While the description of a fracture is frequently correctly included in the report text, the translation of the imaging information into an accurate classification of a fracture is more challenging. Thus, appropriate usage of classification systems is highly dependent on experience^1^ and hampered by the parallelity of various classification systems^2^.

Artificial intelligence (AI)-based algorithms employing large language models (LLM) can address this by comprehending and interpreting human language. OpenAI introduced ChatGPT to a wide audience in November 2022. This chatbot, specifically trained for conversation, is based on a generative pre-trained transformer and the latest iteration GPT 3.5-Turbo. ChatGPT and especially using GPT 4 (released in March 2023, with at the moment still limited access) were shown to provide substantial medical knowledge being able to pass the United States Medical Licensing Examination (USMLE)^3, 4^.

ChatGPT enables rapid processing of complex information and its potential applications in clinical radiology routines have been extensively explored and published in preprints. These comprise the preparation of radiological reports^5^, transferring radiological reports into plain language by simplifying them^6, 7^, or providing clinical decision support on differential diagnoses, diagnostic procedures, final diagnosis, and treatment^8^.

However, ChatGPT is limited to its training data (GPT 3.5-Turbo and GPT 4 trained on data up to September 2021) and thus may not have access to the latest and most specialized knowledge, or might be biased due to the large number of different sources of training data. Therefore, ChatGPT could provide incorrect or incomplete information.

Recently, the feasibility of enhancing ChatGPT’s performance to produce recommendations according to imaging appropriateness guidelines was demonstrated by providing specialized knowledge to the chatbot^9^. For this, data was structured via the LlamaIndex python library. This allows for connecting this specific data to a general LLM in a modular fashion.

We sought to create an AO classification criteria context-aware chatbot (FraCChat) to provide accurate fracture classification based on the report text. To explore this approach, we benchmarked radiologists and publicly available generic chatbots based on GPT 3.5-Turbo and GPT 4 against the FraCChat chatbot built upon GPT 3.5-Turbo and GPT 4 and enhanced with knowledge of the AO classification criteria using a vectorized knowledge index.

## Methods

**Figure 1:**
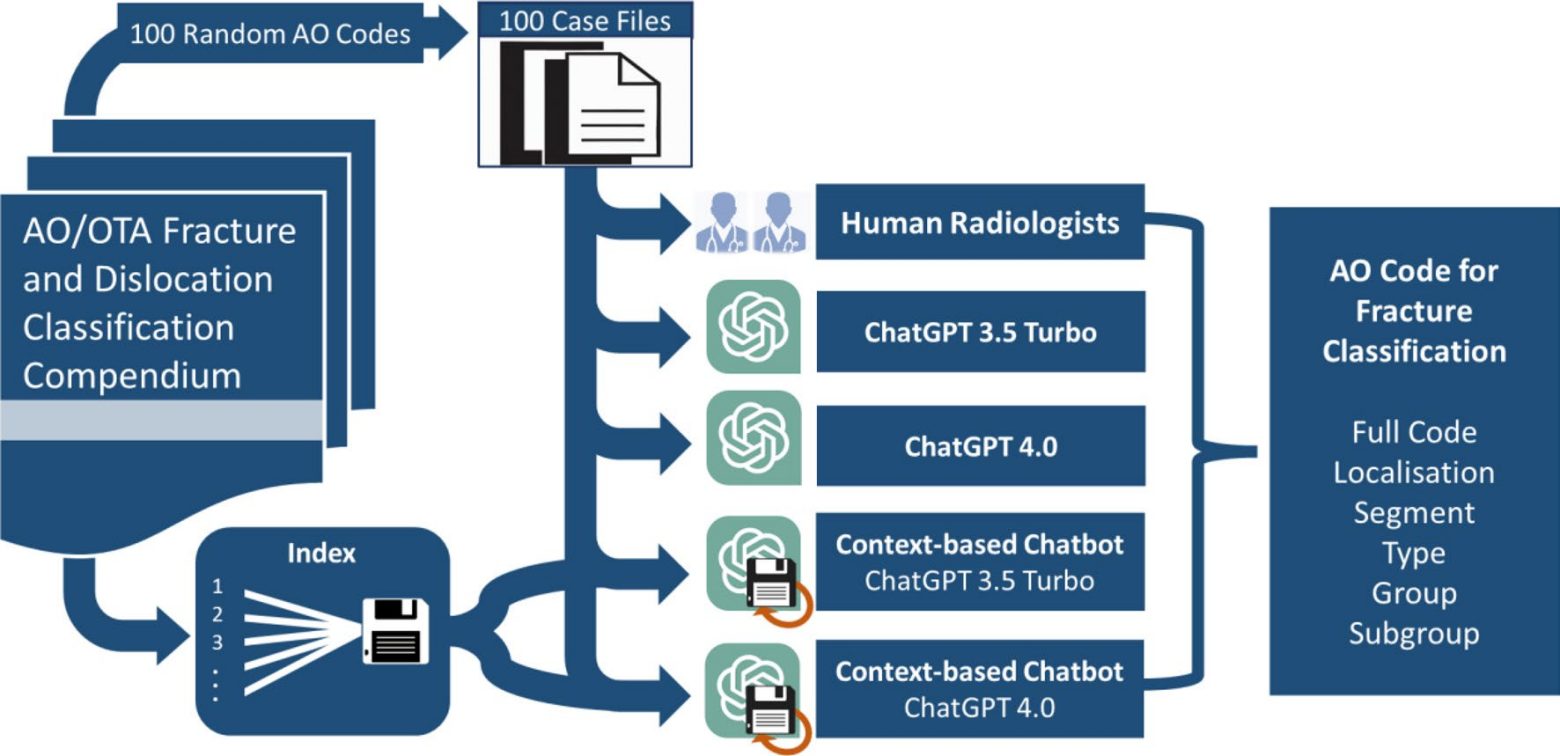
Schematic of the workflow of the case file creation, indexing of the context-aware chatbot and performance analysis. AO/OTA—Arbeitsgemeinschaft Osteosynthese/Orthopaedic Trauma Association.

A schematic of the study workflow is depicted in Fig. 1.

### Technical implementation of the chatbots

#### Data preparation and indexing

To develop and evaluate the proposed FraCChat chatbots, we relied on the AOOTA Fracture and Dislocation Classification Compendium—2018 as a foundational knowledge base.

We employed LlamaIndex as an interface between external data and the GPT 3.5-Turbo or GPT 4 (https://github.com/jerryjliu/llama_index Version 0.5.0). Text information was extracted from the classification compendium and an index was created using the GPTVectorIndex function of LlamaIndex. For this, the document text was divided into smaller chunks of up to 512 tokens (a measure of text content) and converted to data nodes. These nodes were then encoded using an embedding model (text-embedding-ada-002-v2 by OpenAI) and the result was stored in a dictionary-like structure.

#### Prompting strategy and answer synthesis

To customize the output of all chatbots for the case-based scenarios, the question posed to the system in each case was: *"Examine the given fracture description and identify the AO Classification up to Subgroups. Begin your response always with "Full AO Code:"*

The direct output of GPT 3.5-Turbo and GPT 4, was captured as the response. For our context-based chatbot, a multistep answer creation and refinement method was used. As a first step, the case description was transferred to an embedding using the same embedding model (text-embedding-ada-002-v2 by OpenAI) and the five best matching data nodes from the index were retrieved. These nodes were used in the answer creation method using either GPT 3.5-Turbo or GPT 4. As a new input the task based on the given prompt, the case and the references where presented as new input to the language model with an overarching prompt to answer the given task based on the presented information. The final output was then captured.

### Preparation of case files

The chatbots’ accuracy in comparison to human performance was tested in a scenario resembling clinical routine. For this, 100 radiological reports describing fractures were created. From the more than 500 options of the AO classification (neglecting qualifications and universal modifiers), we randomly chose 100. This resulted in 14 cases for humerus, 1 for scapula, 4 for clavicle, 9 for radius, 4 for ulna, 9 for femur, 3 for patella, 14 for tibia, 7 for tibia/fibula, 3 for fibula, 12 for pelvis, 12 for hand/carpus and 8 for the foot. Groundtruth AO codes for the case files comprised information on bone, segment and type of fracture in all cases while information on group was available in 73% and on subgroup in 56%.

The reports were then created by an experienced resident describing the fracture in a manner sufficient for classification.

### Assessment of human and chatbot performance

The 100 case files were presented to two radiologists and classified independently according to the AO classification. During this assessment consultation of guidelines was allowed.

We utilized a script-based approach on the 100 case files to perform a fivefold repetition testing for all four chatbots. From this, the median of the performance of the five runs was calculated.

The performance of humans and chatbots was assessed through qualitative analysis of the different levels of the AO classification extracted from the full AO code: (I) full AO code, (II) bone location, (III) part of the bone, (IV) type of fracture, (V) group, and (VI) subgroup. To compare the respective outputs of humans and chatbots, we employed a Generalized Linear Mixed Model (GLMM) with a binomial family (logit link) to investigate the relationship between the dependent variable "rating" and the independent variable "method." The rating method (human, GPT 3.5-Turbo, GPT 4, FraCChat 3.5 and FraCChat 4) was included as a fixed factor, while allowing for random intercepts for the „case “ and the „rater “.

The consistency of the chatbots was evaluated by comparing the proportion of correct answers that were provided consistently across all five runs versus inconsistently provided correct answers. For this, we calculated the ratios and employed one-sided chi-squared test to assess whether more consistent correct answers were provided than inconsistent ones.

### Assessment of time-effectiveness of radiologists and chatbots

To investigate time-effectiveness, we investigated the time to decision per human reader and chatbot and compared this duration via student’s t-test.

## Results

### Human and chatbot performance

Both radiologists (with access to the AO classification documents) classified the reports according to the AO with high accuracy as they both provided the correct full AO code in 95% of the cases and even higher proportions of correct classifications were noted for the location, fracture type and group.

In contrast, the chatbots generally performed inferiorly as given in Tables 1 and 2. Here, the generic chatbots GPT 3.5-Turbo and GPT 4 only provided correct full AO codes in 5 and 7% of the cases. Better performance was noted for the FraCChat based on GPT 3.5-Turbo (57% correct full AO codes) while the FraCChat relying on GPT 4 reached 83% correct full AO codes. This was corroborated by the performance in the localisation, type and subtype were we noted a similar gradient, too, with mostly significantly better performance of FraCChat 4 over FraCChat 3.5 over GPT 4 over GPT 3.5-Turbo (please see Table 2 for more details). Of note, the performance of FraCChat 4 was not inferior to human rating regarding location, part of bone, fracture type and group as presented in Table 2.

**Table 1:** Performance of human experts and chatbots in AO classification tasks and respective time consumption.

In n cases	Full AO	Location	Part of bone	Type	Group	Subgroup	Time		
	100	100	100	100	73	56	Total (min)	Mean (s)	Range (s)
Human 1	0.95	0.98	0.99	0.98	0.96	0.93	67.5	40.5	7–179
Human 2	0.95	0.98	0.98	0.98	0.96	0.96	98.9	59.4	22–115
GPT 3.5 Turbo	0.05	0.52	0.59	0.22	0.15	0.05	2.7	1.6	0.8–7.2
GPT 4	0.07	0.61	0.68	0.34	0.17	0.02	4.0	2.4	1.3–9.3
FraCGPT 3.5	0.57	0.91	0.96	0.88	0.75	0.42	5.8	3.5	1.3–33.3
FraCGPT 4	0.83	0.97	0.99	0.97	0.9	0.79	8.7	5.2	2.1–13.1

**Table 2:** Detailed comparison of the performance of humans and chatbots accross the different levels of the AO classification system.

	GPT 3.5-Turbo	GPT 4	FraCChat 3.5	FraCChat 4	Human
Full AO					
GPT 3.5-Turbo	–	−0.44 (0.64)	−4.79 (< 0.001)	−7.17 (< 0.001)	−9.20 (< 0.001)
GPT 4	0.44 (0.64)	–	−4.36 (< 0.001)	−6.73 (< 0.001)	−8.77 (< 0.001)
FraCChat 3.5	4.79 (< 0.001)	4.36 (< 0.001)	–	−2.37 (< 0.001)	−4.41 (< 0.001)
FraCChat 4	7.17 (< 0.001)	6.73 (< 0.001)	2.37 (< 0.001)	–	−2.04 (< 0.001)
Human	9.20 (< 0.001)	8.77 (< 0.001)	4.41 (< 0.001)	2.04 (< 0.001)	–
Location					
GPT 3.5-Turbo	–	−1.49 (< 0.001)	−6.71 (< 0.001)	−9.01 (< 0.001)	−9.96 (< 0.001)
GPT 4	1.49 (< 0.001)	–	−5.22 (< 0.001)	−7.52 (< 0.001)	−8.47 (< 0.001)
FraCChat 3.5	6.71 (< 0.001)	5.22 (< 0.001)	–	−2.31 (< 0.001)	−3.26 (< 0.001)
FraCChat 4	9.01 (< 0.001)	7.52 (< 0.001)	2.31 (< 0.001)	–	−0.95 (0.70)
Human	9.96 (< 0.001)	8.47 (< 0.001)	3.26 (< 0.001)	0.95 (0.70)	–
Part of bone					
GPT 3.5-Turbo	–	−1.33 (< 0.001)	−7.03 (< 0.001)	−9.99 (< 0.001)	−9.23 (< 0.001)
GPT 4	1.33 (< 0.001)	–	−5.69 (< 0.001)	−8.66 (< 0.001)	−7.90 (< 0.001)
FraCChat 3.5	7.03 (< 0.001)	5.69 (< 0.001)	–	−2.97 (0.002)	−2.21 (0.11)
FraCChat 4	9.99 (< 0.001)	8.66 (< 0.001)	2.97 (0.0021)	–	0.76 (0.95)
Human	9.23 (< 0.001)	7.90 (< 0.001)	2.21 (0.11)	−0.76 (0.95)	–
Type					
GPT 3.5-Turbo	–	−0.88 (< 0.001)	−6.00 (< 0.001)	−8.56 (< 0.001)	−9.39 (< 0.001)
GPT 4	0.88 (< 0.001)	–	−5.13 (< 0.001)	−7.68 (< 0.001)	−8.52 (< 0.001)
FraCChat 3.5	6.00 (< 0.001)	5.13 (< 0.001)	–	−2.56 (< 0.001)	−3.39 (< 0.001)
FraCChat 4	8.56 (< 0.001)	7.68 (< 0.001)	2.56 (< 0.001)	–	−0.83 (0.75)
Human	9.39 (< 0.001)	8.52 (< 0.001)	3.39 (< 0.001)	0.83 (0.75)	–
Group					
GPT 3.5-Turbo	–	−0.72 (0.02)	−4.19 (< 0.001)	−5.79 (< 0.001)	−7.04 (< 0.001)
GPT 4	0.72 (0.02)	–	−3.47 (< 0.001)	−5.07 (< 0.001)	−6.32 (< 0.001)
FraCChat 3.5	4.19 (< 0.001)	3.47 (< 0.001)	–	−1.59 (< 0.001)	−2.85 (< 0.001)
FraCChat 4	5.79 (< 0.001)	5.07 (< 0.001)	1.59 (< 0.001)	–	−1.25 (0.12)
Human	7.04 (< 0.001)	6.32 (< 0.001)	2.85 (< 0.000)	1.25 (0.12)	–
Subgroup					
GPT 3.5-Turbo	–	0.09 (0.99)	−3.47 (< 0.001)	−5.89 (< 0.001)	−7.96 (< 0.001)
GPT 4	−0.09 (0.99)	−	−3.56 (< 0.001)	−5.99 (< 0.001)	−8.05 (< 0.001)
FraCChat 3.5	3.47 (< 0.001)	3.56 (< 0.001)	−	−2.42 (< 0.001)	−4.49 (< .001)
FraCChat 4	5.89 (< 0.001)	5.99 (< 0.001)	2.42 (< 0.001)	–	−2.07 (< 0.001)
Human	7.96 (< 0.001)	8.05 (< 0.001)	4.49 (< 0.001)	2.07 (< 0.001)	–

Analysis of consistency revealed a higher proportion of consistently correct answers for the context-aware chatbots across all investigated levels of the AO code. Further, we observed that context-aware chatbots did provide significantly more consistently correct answers for the levels type and subgroup, while generic chatbots did not. Details are provided in Table 3.

**Table 3:** Number of correctly provided AO codes and proportion of consistently correct answers.

AO Code	In n cases		Human 1	Human 2	GPT 3.5	GPT 4	FraCChat 3.5	FraCChat 4
Full AO	100	Correct	95	95	5	7	57	83
		Consistent correct answers (%)			3%	2%*	48%***	71%***
Location	100	Correct	98	98	52	61	91	97
		Consistent correct answers (%)			42%***	56%***	88%***	96%***
Part of Bone	100	Correct	99	98	59	68	96	99
		Consistent correct answers (%)			46%***	63%***	93%***	99%***
Type	100	Correct	98	98	22	34	88	97
		Consistent correct answers (%)			13%	20%	83%***	96%***
Group	73	Correct	70	70	11	12	55	66
		Consistent correct answers (%)			8%	11%*	67%***	82%***
Subgroup	56	Correct	52	54	3	1	24	44
		Consistent correct answers (%)			2%	0%	36%*	59%***

Outputs were tested whether the proportion of consistently provided correct answers was higher than inconsistently provided correct answers by one-sided chi squared test. Results are given with * for p < 0.05 and *** for p < .001.

### Time effectiveness of radiologists and chatbots

Duration to provide answers on the 100 case files was substantially lower for the generic chatbots than for human readers (generic chatbot mean 3.3 min vs. human mean 83.2 min; p < 0.001). In addition, generic chatbots were significantly faster than context-aware chatbots (generic chatbot mean 3.3 min vs. context-aware chatbot mean of 7.2 min; p < 0.001) and chatbots based on GPT 3.5 were both faster than the respective GPT 4 based one (both p < 0.001).

## Discussion

In this study, we assessed the feasibility of generic and context-aware chatbots to provide AO fracture classification codes from radiological reports. Though the time needed to provide AO classification was significantly lower for chatbots than humans, we observed an inferior performance compared to human radiologists. However, we found that providing specific knowledge substantially enhances the chatbot’s performance and consistency and not being inferior to human rating in terms of location, part of bone, fracture type and group.

Various AI approaches have been proposed for fracture detection based on the image dataset, reaching to whole-body approaches as commercially available tools^10^. AI based methods are also available for image-based fracture classification, but mostly are limited to a particular region of the body. For example, for the ankle^11^, the distal radius^12^, the hip^13^ and the knee^14^, convincing results were obtained by deep learning and the respective AO classifications derived directly from the image dataset.

Our approach follows a different track—human radiologists are easily capable of adequately describing a fracture in terms of its morphology and complexity. The translation of this description into the respective AO code classification was performed by an AI approach in this proof-of-concept work. Although we did not reach the performance of a human classifier on all levels of the AO system but only for location, part of bone, fracture type and group, we were able to show that chatbots based on LLM are very well suited for this purpose and might improve workflows as they are more time efficient than human radiologists. In addition, the provision of specialized knowledge showed a clear superiority over generic chatbots; not only the performance but also the consistency were substantially improved.

Previous studies employed natural language processing (NLP) to extract data from radiology reports. Good performance was found in NLP algorithms that were specifically trained to extract fracture presence^15^ or fracture site^16^. Deep learning-based NLP methods using Convolutional Neural Networks have shown improved performance compared to classical NLP approaches. However, these approaches require rather large datasets as the performance scales with the amount of training data as shown for classification of hip fractures or smoking status^17^. In contrast to those specifically trained models, the method of providing specific knowledge via a vector index to a pretrained generic LLM comes with several advantages. The LlamaIndex interface offers a flexible data connector between an existing data source and LLM, structuring and optimizing data. Through this, the specific knowledge base can be exchanged swiftly and independently of the original LLM, removing the need of a task specific trained or fine-tuned model. Furthermore, the generated index is editable and individual references can be added or deleted (for example, if new guidelines are published or amended). In return, an existing index can be provided to a new LLM as content, avoiding the need for resource-consuming re-training of the AI algorithm itself.

The significantly higher performance of chatbots based on GPT version 4 compared with 3.5-Turbo is corroborated by recent studies on the simplification of radiological reports^7^ or passing United States Medical Licensing Examination questions^4^. Further improvement in performance is expected with newer GPT models and better vector indices as we noted a significant increase in performance and consistency for GPT 4 based generic and context-aware chatbots over the GPT 3.5-Turbo based variants. This might also address the limitation we encountered with all chatbots in the case of the complex hindfoot fracture (AO 89A). Here, the chatbots mostly correctly classified the individual fractures of the talus and the calcaneus, but did not manage the translation to AO 89A. An interesting pattern was observed in the case file for AO 2U3A3. Despite the description of "multiple bone fragments", the chatbots incorrectly classified 2U2 whereas the human raters assigned the correct AO code.

Potential applications of the presented approach go beyond just supporting AO classification in clinical routine, but extent to retrospective analyses of existing reports and comparison with further procedures of the patients for quality control. In addition, the extension to other classification systems is also conceivable as well as providing information on evidence-based therapies with provision of the respective evidence in clinical routine. Nevertheless, this approach requires validation with real-world case files to test the generalizability.

In conclusion, we found that a context-aware chatbot can derive structured information on fracture classification from radiological reports. Though not reaching human performance, the time consumption was significantly lower.

We nevertheless provide evidence that refining and providing specific context to ChatGPT will be the next essential step in harnessing its power.

## Data Availability

The datasets generated during and analyzed during the current study are available from the corresponding author on reasonable request.

## Code availability

The code for recreating the presented FraCChat chatbot was made available as an interactive jupyter notebook on an open Git repository (https://github.com/maxrusse/fracchat). Further code used in our analysis is available from the corresponding author upon reasonable request.
